# Midwifery Students’ Views on NANDA-I Diagnoses and Care Plans Used in Clinical Practice

**DOI:** 10.3390/healthcare12121196

**Published:** 2024-06-14

**Authors:** Leyla Baran, Yeşim Yeşil

**Affiliations:** 1 Faculty of Health Sciences, Mardin Artuklu University, 47100 Mardin, Turkey; 2 Department of Midwifery, Faculty of Health Sciences, Mardin Artuklu University, 47100 Mardin, Turkey; yesim.yesil89@gmail.com

**Keywords:** midwifery, students, midwifery education, care plan, NANDA-I

## Abstract

Background: Developing a care plan for patients is not specific to nursing or midwifery; it is the case in every situation where patients seek help from care providers. Midwifery students draw upon NANDA-I diagnoses while developing care plans (CPs) in clinical practices, and plan and apply their care accordingly. This study aims to identify the views of midwifery students on the NANDA-I diagnoses and CPs that they use in clinical practice. Methods: This descriptive and cross-sectional study was conducted with 222 students between September and December 2022. The research data were collected through face-to-face interviews using a questionnaire based on NANDA-I diagnoses and CPs. In line with the existing literature, the questionnaire was designed by two academicians who specialized in nursing fundamentals and midwifery. The questionnaire consisted of closed-ended (8 questions) and open-ended (7 questions) questions. Data analysis was performed with descriptive statistics and Pearson’s chi-square test. Results: Of 222 students, 57.7% stated that they did not know the definition of a CP. It was found that 46.8% of the students felt inadequate at developing CPs. Furthermore, the students stated that they found the CP training provided during their undergraduate study partially sufficient, with a rate of 52.7%, and 16.7% of midwifery students offered suggestions about CP teaching. The number of midwifery-related diagnoses of which the students were aware was 19. Conclusions: The study was beneficial in terms of identifying the deficiencies of the students related to NANDA-I diagnoses and developing CPs that might be overcome through education and observing the midwifery-related diagnoses that they used. It is of critical importance to educate midwifery students about midwifery-related NANDA-I diagnoses in order that they embrace midwifery diagnoses and CP learning.

## 1. Introduction

The International Confederation of Midwives (ICM) bases its efforts to expand the number and capacity of midwives and strengthen midwifery globally on three pillars, one of which is midwifery education [1]. ICM emphasizes that a qualified midwifery education should be based on adequacy and clinical practice rather than an academic degree, and it is important to improve the abilities of critical thinking, clinical decision-making, and problem-solving [2]. In “Essential Competencies for Midwifery Practice”, published by ICM in 2019, competencies were arranged in a framework under four interrelated categories: general competencies, competencies specific to prepregnancy and antenatal care, competencies specific to care during labor and birth, and competencies specific to the ongoing care of women and newborns. General competencies are related to care activities which are relevant for all aspects of midwifery practice. Midwifery education in Turkey is delivered within 4-year undergraduate programs on a full-time basis in line with the criteria of the WHO and the EU and includes at least 4 years’ theoretical and practical training [2,3]. Although there are global standards along with regional accreditation criteria, midwifery education does not have a standard accreditation system on a global scale [4]. Midwifery education in Turkey is arranged and implemented in line with the MNCEP (Midwifery National Core Education Program). On the other hand, the scope of fundamental midwifery practices in Turkey has been built on ICM’s international definition of midwife [5]. At Mardin Artuklu University, the midwifery fundamentals course is delivered in the first year of 4-year midwifery education. This course aims to upskill students in order that they acquire basic abilities and train them in providing individual care. In this context, the midwifery fundamentals course includes teaching of care plans (CPs) and NANDA-I diagnoses as well. 

The nursing process/CP involves the following stages: data collection to assess a patient’s status, determination of appropriate diagnoses in line with the data collected, planning interventions that can be applied for the diagnoses determined, implementation of the planned interventions, and evaluation of the results [6]. As they offer high-quality and reliable care to patients and their families, nurses and midwives are at the core of care provision [7]. 

There is a need for to establish a standard language to facilitate communication among healthcare professionals about patients’ health problems [8]. Using a standard terminology in care ensures understanding of patient needs in the same way [9,10]. Providing evidence-based nursing diagnoses, NANDA International (NANDA-I) facilitates the development, improvement, popularization, and utilization of standardized nursing diagnosis terminology and contributes to patient safety by incorporating evidence-based terminology into clinical practice and decision-making [11]. Nursing diagnosis is a clinical nursing decision on the reactions of individuals, families, groups, or society to existing or potential health problems [12,13]. Woman-centered care, which emphasizes the unique individual needs, expectations, and aspirations of women, is often associated with midwife-led care [14]. Midwifery students draw upon NANDA-I diagnoses while developing CPs in clinical practices and plan and apply their care accordingly [15]. Utilization of nursing diagnoses contributes to nurses providing better CPs by enhancing the quality of nursing interventions and patient outcomes [13]. 

In Turkey, although the term “nursing diagnosis” is used in both the education of nursing students and the development of CPs for patient care in hospitals, a term like midwifery diagnosis is not used in the education of midwifery students or in CPs that midwives develop for patient care. In most parts of the world, formal education of professional midwives has been influenced by historical, cultural, and ideological factors. Approaches to education have reflected contemporary debate around the role of midwifery as well as potential conflicts and synergies with medicine and nursing, leading to a marked divergence between countries. For instance, some countries such as the United Kingdom (UK) repositioned midwifery as a nursing specialism, where midwifery education was at times focused on post-nursing programs with an emphasis on pathology and ill health [16]. In contrast, countries like France and Turkey maintained the autonomous role of the midwife and developed direct entry education [3,17].

However, there is a clear need for the classification of midwifery diagnoses. For instance, Ayerle et al. (2019) reported that in German-speaking countries, midwifery diagnoses were not systematized under any classification, and no university with a midwifery curriculum emphasized its importance and taught any midwifery diagnosis practice. In addition, they highlighted that the lack of formulation of midwifery-related diagnoses would be one of the greatest challenges in the future for universities with midwifery undergraduate and postgraduate programs and for the midwifery profession itself [18].

In this context, our study aims to identify midwifery students’ opinions on the NANDA-I diagnoses and CPs that they use in clinical practice. 

## 2. Materials and Methods

### 2.1. Design and Setting 

This study was conceptualized and designed as descriptive and cross-sectional quantitative research and conducted between September 2022 and December 2022. In medical research, “cross-sectional study” refers to a sort of observational study design that includes analysis of data obtained from a population within a specific time period [19]. The study was carried out in the Department of Midwifery, Faculty of Health Sciences at a state university in the Southeastern Anatolia region of Turkey. 

The Department of Midwifery has been producing graduates since 2014 within the Faculty of Health Sciences, founded in 2009. In addition to midwifery, the faculty has the following departments: nursing, nutrition and dietetics, physiotherapy and rehabilitation, pediatric development, social service, audiology, and healthcare management. 

### 2.2. Sampling

The population for the research consisted of 2nd-year (n = 90), 3rd-year (n = 88), and 4th-year (n = 83) students (N = 261) studying in the midwifery department at a state university in the Southeastern Anatolia region of Turkey. Convenience sampling was used to recruit participants. Each student answered the questions alone and independently so as to prevent any possibility of bias. The research sample consisted of 222 students who met the inclusion criteria and agreed to participate (participation rate: 85.3%). According to the formula for a finite (known) population [n = (N.t2.p.q)/(d2.(N − 1) + (t2.p.q)] [N = number of individuals in the target population, n = number of individuals taken as a sample, p = frequency of incident examined (possibility of realization), q = infrequency of incident examined (possibility of nonrealization), t = at a certain significance level, theoretical values in accordance with the *t* table, d = sampling error accepted according to the frequency of incident], sample size was calculated to be 156 with a 95% confidence interval and 5% margin of error [20]. The total number of participants in the study met the sample size requirement. 

*Inclusion criteria were as follows:*

- To be a 2nd-year, 3rd-year or 4th-year student in the midwifery department;
- To have successfully completed the midwifery fundamentals course that includes CP teaching.

*Exclusion criteria were as follows:*

- Students who, in their educational background, received training within a program like nursing which had included a course covering CPs;
- Students who participated in the pilot study (n = 15).

### 2.3. Data Collection

The study data were collected in a classroom environment using a questionnaire through face-to-face interviews between September 2022 and December 2022. Students were given a brief explanation about the purpose of the study before dissemination of the questionnaire forms and were reassured that their data would be confidential and that they could withdraw if they wished. Developed by the researchers in line with the literature, a questionnaire containing 15 questions was used to collect data. The reason why the research team developed the questionnaire was that there was no instrument in the literature to assess midwifery students’ views on NANDA-I diagnoses and CPs. 

*Questionnaire:* In addition to demographic data, such as age and grade, the questionnaire consisted of questions about whether students were aware of the definition of a CP, found the CP training provided within the midwifery fundamentals course during their undergraduate study adequate, and thought of themselves as adequate in terms of planning the care. Furthermore, it had questions aiming to investigate whether there was a need for additional training on pre-clinic CPs and whether they had any suggestions about CP training, and to explore their knowledge about the midwifery-related NANDA-I diagnoses that they had learned and frequently used [13,21,22]. Five academicians who specialized in the Department of Nursing fundamentals gave expert opinions on the questionnaire. Moreover, a pilot study was conducted with five students from each grade (15 in total) in an attempt to evaluate the intelligibility of the questions, and these students were not included in the study. The questionnaire was finalized in this way. 

### 2.4. Data Analysis

The research data were analyzed with “The Package for Social Sciences (SPSS)” software package, Version 22.0 (SPSS Inc., Chicago, IL, USA). Since the participants’ data consisted of categorical variables, number and percentage distributions were used as descriptive statistics. The Pearson chi-square test was used to compare CP knowledge levels and thoughts between the grades. The level of significance was *p* < 0.05. 

### 2.5. Ethical Considerations 

For the research to be carried out by the researchers, an ethics committee permit (nr: 2022-6, dated 8 March 2022) was obtained from Mardin Artuklu University’s Non-Invasive Clinical Research Ethics Committee. Participants were informed beforehand about the study, and their written consent was obtained in line with privacy principles and the Helsinki Declaration.

## 3. Results

A total of 222 students participated in the research. The average age of the students was 20.80 ± 1.46 (min–max: 18–29), and 81 of them (36.5%) were 2nd-year, 73 (32.9%) 3rd-year, and 68 (30.6%) 4th-year (n = 83) students. Students stated that their first CP was taught as a “nursing CP”, with a rate of 81.5%. Only 15.8% stated that midwifery and nursing CPs have the same meaning. When asked about the definition of a CP, 42.3% of the students (n = 94) expressed that they knew it; however, only 34 students could provide a correct CP definition. The students reported that they found CP training given during their undergraduate study partially adequate, with a rate of 52.7%, and they felt inadequate at planning the care, with a rate of 46.8%. 78.8% of the students thought that there was a need for refresher training in CPs and diagnosis each term before hospital practices, whereas 21.2% stated that it was not necessary. When asked about whether they could make suggestions on CP teaching, 16.7% of the students (n = 37) expressed that they could (Table 1). Their suggestions are grouped and presented in Table 2. 

With respect to the question about whether they were taught midwifery-specific diagnoses, 16.7% of the students responded “Yes”, 77% responded “No”, and 6.3% stated they did not recall such a teaching. Those who responded “Yes” were then asked about these diagnoses, and their answers along with NANDA-I diagnoses that the students knew and frequently used are shown in Table 3. 

## 4. Discussion

This study aims to identify the views of midwifery students on the NANDA-I diagnoses and CPs that they use in clinical practice. Midwifery and nursing education in Turkey are offered as two different disciplines at a 4-year undergraduate level of study. In midwifery education, students are taught the development of a CP and nursing diagnoses in the midwifery fundamentals course at the first grade. However, the curriculum might differ since midwifery education is not accredited. In addition, there is no postgraduate-level, department-based branch for midwifery. Therefore, midwifery fundamentals courses are usually delivered by academicians from nursing departments who specialize in the field of nursing fundamentals. CPs are basically the same in midwifery and nursing disciplines, but in our research only 15.8% of the students stated that the midwifery and nursing CP had the same meaning, which implies that the abovementioned reasons led the students to think in this way. 

The CP is an important part of hands-on training in both nursing and midwifery education [21,23], and students are expected to acquire the ability to use CPs during the training. Almost half of the students in the study (46.8%) stated that they felt inadequate in planning care (Table 1). 

Table 2 lists students’ recommendations on CP teaching. There is no study in the literature about the use of care plans and NANDA-I diagnoses by midwifery students. Thus, research findings are discussed in relation to studies conducted with nursing students. Şendir et al. (2009) reported that, like midwifery students, nursing students also had some difficulties in developing and implementing a CP [24]. In another study, students stated that they had difficulty in expressing patients’ care needs as nursing diagnoses [25]. It is of critical importance that a CP should be used in clinical settings so as to make sure that all of its phases function effectively [26]. Vizeshfar and Torabizadeh (2018) suggest that employing the teaching methods that nursing students prefer promotes learning, ensures its permanence, and enhances academic performance [27]. Narration is reported to be the most frequently used method in CP teaching [22]. On the other hand, Thistlethwaite et al. (2012) suggest that case-based learning is an inquiry-based pedagogical approach that prepares students for clinical practice [28]. Similarly, a study analyzing the clinical decision-making abilities of midwifery students reported that case-based teaching was efficient for students in pre-clinical training and used frequently [29]. Case-based teaching is the most frequently suggested method by the students for CPs and it is also underpinned by the literature. It is thought that students use more easily in clinical practice the CPs and diagnoses that they learn on a case. 

Midwifery diagnoses ensure the management to term of an individual or family health problem by means of a clinical evaluation and are regarded accordingly as the basis of professional intervention by the midwife. They also make a significant contribution to the standardization of technical language and enhancement of midwives’ knowledge on their broad and complex practices [30]. In this research, 77% of the students reported that they had not been taught midwifery-specific diagnoses. No study was found in the literature on whether midwifery students were aware of midwifery-related diagnoses. However, NANDA-I has numerous diagnoses related to midwifery [6]. 

For freshmen and sophomores, this lack of awareness might be explained by the following reasons: the midwifery fundamentals course is basically delivered by academicians of nursing origin; courses in the first year predominantly cover fundamental skills and care (injection practices, oral care, etc.); specialized field lectures begin to be delivered in the second year, and, thus, CPs and nursing diagnoses are not taught as specific to midwifery since there is no clinical application related to midwifery. For 3rd-year and 4th-year students the reason might be that teaching academicians in this field are not as qualified as those from the field of nursing fundamentals in terms of CPs and diagnoses. In this sense, no study was found in the literature analyzing the competence of academicians delivering midwifery teaching on diagnoses. 

As seen in Table 3, it was determined that there was a total of 37 different NANDA-I diagnoses that the students knew, frequently used, and thought of as midwifery-specific. NANDA-I has some diagnoses directly and indirectly related to midwifery, which can be called “Midwifery-specific Diagnoses”. *Labor pain*, *ineffective childbearing process*, and *risk for disturbed maternal–fetal dyad* are examples of directly related diagnoses, and *constipation*, *fatigue*, and *risk for unstable blood pressure* are examples of indirectly related diagnoses [6]. However, the reason why the students in our study reported a very low number and variety of diagnoses might be that they thought NANDA-I diagnoses were solely associated with nursing, which is underpinned by the finding that 84.2% of the students thought midwifery and nursing CPs were different (Table 1). 

The set of NANDA-I diagnoses is a classification that can be used by midwives as well, but designating them as “nursing diagnoses” is presumed to lead midwives and midwifery students not to embrace and use them. There are studies in the literature in which NANDA-I nursing diagnoses are used in the areas where midwives work intensively, such as postnatal service, prenatal period, and delivery room [31,32,33]. However, there is no study in the literature on nursing diagnoses used by midwifery students, and only one study has been found about nursing diagnoses that midwives use [34]. 

The research is limited to the department of midwifery of the state university in which the study was conducted, and, hence, the results cannot be generalized. Ideally, it should be repeated with wider sample groups.

## 5. Conclusions

NANDA-I diagnoses, which are learned by nursing students during their education and used by nurses as one of the steps of the care plan while planning patient care, increase the quality of care by creating a standard language in nursing. In healthcare, midwives, like nurses, are among the health personnel who spend the most time with the patient. In countries where midwifery and nursing programs are separate programs, the use of NANDA-I diagnoses as “nursing diagnoses” in the usage language may cause midwifery students and, therefore, midwives not to adopt and not use NANDA-I diagnoses as a care plan step.

In the midwifery curriculum, training on nursing diagnoses and developing CPs is delivered solely within the midwifery fundamentals course in the first year of study. In this context, as the students also stated, it might be suggested that students should receive refresher training on these areas each term before clinical practice in order that they feel more competent. Teaching the diagnoses as “nursing diagnoses” in the lectures is thought to cause midwifery students not to internalize NANDA-I diagnoses. Therefore, lecturers in midwifery should be aware of this situation and properly state that the use of NANDA-I diagnoses also covers midwifery practices. Furthermore, it might be recommended that they teach CPs and diagnoses through methods enabling students to communicate and interact with each other, such as group discussions and brainstorming. Moreover, pre-clinic case-based teaching in particular is thought to facilitate students’ understanding and comprehension of these subjects.

## Figures and Tables

**Table 1:** Students’ knowledge levels and thoughts on the care plan.

Variables	2nd Grade (n = 81)		3rd Grade (n = 73)		4th Grade (n = 68)		Total (N = 222)		*p* *
	n	%	n	%	n	%	n	%	
When the “Care Plan” was first taught, it was taught as “Nursing Care Plan”.									
Yes	67	82.7	52	71.2	62	91.2	181	81.5	0.009
No	14	17.3	21	28.8	6	8.8	41	18.5	
Do “Midwifery Care Plan” and “Nursing Care Plan” have the same meaning?									
Yes	17	21.0	12	16.4	6	8.8	35	15.8	0.125
No	64	79.0	61	83.6	62	91.2	187	84.2	
Do you know the definition of “Care Plan”?									
Yes	34	42.0	32	43.8	28	41.2	94	42.3	0.947
No	47	58.0	41	56.2	40	58.8	128	57.7	
Do you find the “Care Plan” teaching given in the undergraduate study sufficient?									
Sufficient	30	37.0	26	35.6	24	35.3	80	36.0	0.969
Partially sufficient	42	51.9	40	54.8	35	51.5	117	52.7	
Insufficient	9	11.1	7	9.6	9	13.2	25	11.3	
Do you see yourself as sufficient in planning care?									
Sufficient	9	11.1	5	6.8	7	10.3	21	9.5	0.293
Partially sufficient	31	38.3	30	41.1	36	52.9	97	43.7	
Insufficient	41	50.6	38	52.1	25	36.8	104	46.8	
Are preclinical “Care Plan” reinforcement trainings necessary?									
Necessary	66	81.5	60	82.2	49	72.1	175	78.8	0.259
Unnecessary	15	18.5	13	17.8	19	27.9	47	21.2	
Do you have any suggestion about “Care Plan” teaching?									
Yes	15	18.5	7	9.6	15	22.1	37	16.7	0.119
No	66	81.5	66	90.4	53	77.9	185	83.3	

* Pearson chi-square test.

**Table 2:** Students’ suggestions about care plan teaching.

Suggestions (Similar Recommendations are Grouped)	n	%
In each preclinical period, sample care plans based on frequently used diagnoses in the clinic should be taught in skill laboratories.	16	43.3
2. Care plans prepared in line with the data collected from the patient should be discussed with the responsible faculty member.	9	24.3
3. More case studies should be given in care plan teaching.	7	18.9
4. The care plan should be taught by discussing numerous and various cases with a small number of students.	2	5.4
5. Different techniques should be used in care plan teaching. (Note: techniques were not mentioned)	2	5.4
6. Care plans to be prepared for the first time should be taught to real patient.	1	2.7
Total	37	100

**Table 3:** Distribution of NANDA-I diagnoses that students know, frequently use, and regard as specific to midwifery.

No.	NANDA-I Diagnoses	Number of Students Who		
		Know	Frequently Use	Regard as Specific to Midwifery
1	Risk for Infection	12	19	4
2	Risk for Adult Falls	15	16	3
3	Diarrhea	16	14	--
4	Anxiety	17	6	3
5	Acute Pain	9	14	3
6	Risk for Electrolyte Imbalance	5	9	2
7	Stress Overload	1	14	1
8	Constipation	7	9	--
9	Excess Fluid Volume	6	5	4
10	Deficient Fluid Volume	8	3	--
11	Impaired Comfort	7	2	2
12	Deficient Knowledge	5	3	1
13	Imbalanced Nutrition: Less than Body Requirements	4	3	2
14	Hyperthermia	2	7	--
15	Disturbed Sleep Pattern	5	3	1
16	Nausea	6	1	1
17	Impaired Tissue Integrity	4	3	--
18	Fatigue	4	1	1
19	Impaired Physical Mobility	2	2	2
20	Risk for Bleeding	2	1	2
21	Risk for Unstable Blood Pressure	2	1	1
22	Decreased Activity Tolerance	2	2	--
23	Risk for Unstable Blood Glucose Level	1	1	1
24	Risk for Contamination	3	--	--
25	Urge Urinary Incontinence	3	--	--
26	Impaired Oral Mucous Membrane Integrity	1	2	--
27	Delayed Surgical Recovery	2	--	--
28	Self-Neglect	2	--	--
29	Hypothermia	1	--	--
30	Risk for Sudden Infant Death	--	--	1
31	Risk for Injury	1	--	--
32	Impaired Social Interaction	1	--	--
33	Ineffective Role Performance	--	--	1
34	Disorganized Infant Behavior	1	--	--
35	Risk for Aspiration	1	--	--
36	Impaired Spontaneous Ventilation	1	--	--
37	Fear	--	1	--
Total Number of Different Diagnoses		35	25	19

## Data Availability

The data presented in this study are available on request from the corresponding author.
